# Visible-Light-Driven Semiconductor–Metal Transition in Electron Gas at the (100) Surface of KTaO_3_

**DOI:** 10.3390/nano13233055

**Published:** 2023-11-30

**Authors:** Xiaochen Tian, Bocheng Li, Hu Sun, Yucheng Jiang, Run Zhao, Meng Zhao, Ju Gao, Jie Xing, Jie Qiu, Guozhen Liu

**Affiliations:** 1 Jiangsu Key Laboratory of Micro and Nano Heat Fluid Flow Technology and Energy Application, School of Physical Science and Technology, Suzhou University of Science and Technology, Suzhou 215009, Chinajyc@usts.edu.cn (Y.J.); zr@usts.edu.cn (R.Z.); mzhao@usts.edu.cn (M.Z.); jugao@hku.hk (J.G.); 2 School of Science, China University of Geosciences, Beijing 100083, China; xingjie@cugb.edu.cn

**Keywords:** KTaO_3_, surface 2DEG, photoelectric response, semiconductor–metal transition

## Abstract

Two-dimensional electron gas (2DEG) at the (100) KTaO_3_(KTO) surface and interfaces has attracted extensive interest because of its abundant physical properties. Here, light illumination-induced semiconductor–metal transition in the 2DEG at the KTO surface was investigated. 2DEG was formed at the surface of KTO by argon ion bombardment. The 2DEG prepared with a shorter bombardment time (300 s) exhibits semiconducting behavior in the range of 20~300 K in the dark. However, it shows a different resistance behavior, namely, a metallic state above ~55 K and a semiconducting state below ~55 K when exposed to visible light (405 nm) with a giant conductivity increase of about eight orders of magnitude at 20 K. The suppression of the semiconducting behavior is found to be more pronounced with increasing light power. After removing the illumination, the resistance cannot recover quickly, exhibiting persistent photoconductivity. More interestingly, the photoresponse of the 2DEG below 50 K was almost independent of the laser wavelength, although the photon energy is lower than the band gap of KTO. The present results provide experimental support for tuning oxide 2DEG by photoexcitation, suggesting promising applications of KTO-based 2DEG in future electronic and optoelectronic devices.

## 1. Introduction

Complex perovskite oxides display many exotic properties, such as high T_C_ superconductivity, large magnetoresistance, ferromagnetism, ferroelectricity [1,2,3], etc. It has been noted that oxygen vacancies in transition-metal oxides play an important role in structural transformation and physical properties [4,5]. In 2004, Ohtomo and Hwang observed a high-mobility two-dimensional electron gas (2DEG) at the interface composed of insulating oxides SrTiO_3_(STO) and LaAlO_3_(LAO) [6]. Subsequently, a variety of fascinating phenomena has been discovered in the 2DEG at this interface, such as superconductivity [7], ferromagnetism [8], charge ordering [9], thermoelectricity [10], Shubnikov-de Haas oscillations [11], quantum Hall effect [12], strong electron correlation [13], etc. Furthermore, 2DEG can also be formed at the surface of STO crystals [14,15]. Unlike the 2DEG at oxide interfaces, 2DEG at the oxide surface can be directly combined with other semiconductors in various ways to form devices with different structures, providing new solutions for future miniaturized multifunctional electronic devices [16,17].

In the perovskite family, KTaO_3_(KTO) is another promising candidate, which has a similar crystal structure, lattice parameters, electron-effective mass, and the width of the band gap as STO [18], so it may also be expected to exhibit rich and attractive physical properties in KTO-based heterojunctions. Moreover, it has a spin-orbit coupling strength of at least two orders of magnitude higher than that of STO [19,20]. Related studies have proved that high-mobility electrons appear at the surface of KTO crystal and the interfaces between KTO film and other oxides [21]. Furthermore, the KTO-based 2DEG shows more amazing properties than that at the STO-based interfaces or surfaces, such as higher superconducting temperatures [22], thermal spin injection, and inverse Edelstein effect [23].

Electric field and light are currently popular methods for manipulating the carrier density of oxide 2DEG [24]. Compared with the electric field, light is a more convenient stimulus in tuning the electronic configuration, and hence the physical properties of a system [25,26,27]. Remarkable photocatalytic properties have been observed in semiconductor-based devices [28,29,30]. The study of light-induced phenomena can help develop devices such as optical switches, photocells, and optical storage for practical applications [31,32]. The illumination of STO-based systems by ultraviolet/visible light has been demonstrated to enhance electrical conductivity by several orders of magnitude [33,34], where oxygen vacancies play an essential role in the photoresponse [35,36,37,38,39]. So far, most of the previous works on KTO-based 2DEG focused on the metallic state [40,41], and there is little information about the effect of visible light photoexcitation on the semiconducting state or insulating state. In addition, the photoconductivity of KTO-based 2DEG has usually been studied at room temperature or several selected temperatures, and it is difficult to know the temperature-dependent photoconductivity based on these scattered data. For example, Goyal et al. investigated the effects of visible light on the 2DEG at the KTO interface under room temperature conditions [42], and Tomar et al. also studied the photoresponse of Ar-bombarded KTO with daylight and visible laser light illumination at room temperature [41]. Although temperature-dependent measurements were carried out on single-crystal KTO (001) by Zeeshan et al., the UV light excitation is a combinational effect of defect-related shallow donors and band–band-related deep donors, making it difficult to understand the roles oxygen vacancies played in the KTO-based surface 2DEG [43].

In the current study, we observed visible light illumination-induced semiconducting-metallic transition in 2DEG at the KTO surface. The as-prepared KTO electron gas shows semiconducting behavior in the whole temperature range of 300–20 K. After 405 nm light irradiation, the semiconducting state is highly suppressed, and it appears only at temperatures below 55 K. The suppression of the semiconducting state is found to be more pronounced with increasing light power. It is expected that the low-temperature semiconducting state would be entirely inhabited if the sample surface is irradiated at a sufficiently high laser power. In addition, different from previous observations in oxide 2DEG [44], the photoresponse of 2DEG at KTO at temperatures below 50 K was almost independent of laser wavelength, indicating a shallow energy level existing below the conduction band minimum (CBM) of KTO.

## 2. Materials and Methods

**Figure 1:**
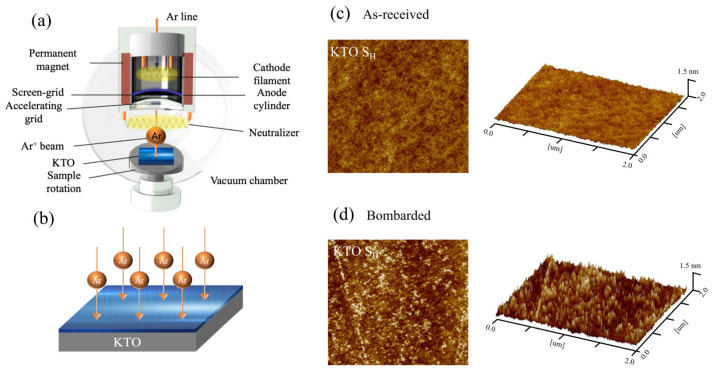
(**a**) Diagram of ion beam bombardment device. (**b**) The preparation process of the 2DEG at the KTO surface. The 2D and 3D surface morphology of sample S_H_ before (**c**) and after (**d**) Ar ion bombardment (2 × 2 μm^2^).

The samples were prepared by an argon-ion-beam-assisted bombardment method (Ar ion bombardment assistant, AIBA) at the KTO surface with a dimension of 5 × 3 mm^2^ at room temperature. The ion beam bombardment device is shown in Figure 1a. The bombardment voltage was 300 V, and the oxygen pressure was 5 × 10^−6^ mbar; the schematic diagram of the preparation process is shown in Figure 1b. Two samples with room temperature resistances of 50 kΩ (sample S_H_, 300 s bombardment time) and 8.6 kΩ (sample S_L_, 480 s bombardment time) were obtained by varying the bombardment duration of the argon ions. Usually, a longer bombardment time will introduce oxygen vacancies with larger numbers in KTO, and the electron concentration in sample S_L_ is higher, but is still much lower than that in traditional metals.

The surface morphology of sample S_H_ was checked by atomic force microscopy (AFM). Figure 1c,d show both the 2D and 3D surface topography before and after bombardment, respectively. Ultrasonic Al-wire bonding was adopted for electrical contacts, and the separation between two neighboring probes was 1 mm. Standard four-probe technique was employed for resistance measurements, and the applied voltage for resistance measurement was 2.5 V. All the measurements were made in a physical property measurement system (PPMS). Before all the measurements, the samples were preserved in the PPMS chamber in the dark for approximately 12 h to eliminate the effects of ambient light. For light illumination measurements, continuous lasers at 405 nm, 447 nm, 532 nm, 655 nm, and 808 nm were introduced into the PPMS with a spot area of 0.11 cm^2^.

## 3. Results and Discussion

### 3.1. Surface Morphology before and after Bombardment

Figure 1b,c show the surface morphology of the as-received and after-bombarded KTO crystal. The surface did not change significantly after it was bombarded, except that the surface root-mean-square roughness increased slightly, which was 0.24 nm and 0.14 nm after and before the bombardment, respectively. This is different from the case in the oxides grown by pulsed laser deposition, which leads to an obvious change in the stoichiometry and crystal structure [45], while the surface 2DEG obtained by ion beam bombardment can well maintain its crystal structure.

### 3.2. Temperature-Dependent Resistance with and without Light

**Figure 2:**
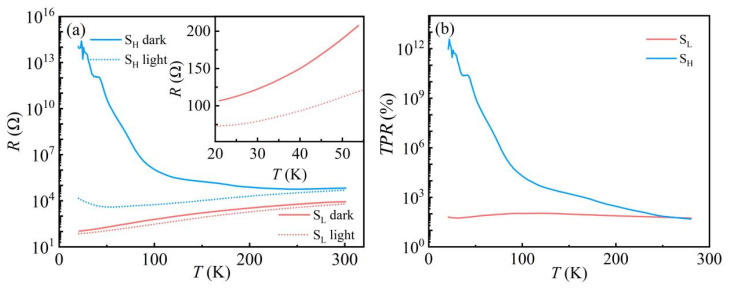
(**a**) Resistance of the 2DEG at the KTO surface in dark and light as a function of temperature; the light wavelength and power are 405 nm and 1.66 μW, respectively. The inset shows the resistance versus temperature curve for sample S_L_ at 20–55 K; (**b**) *TPR* of the 2DEG at the KTO surface as a function of temperature.

Figure 2a indicates the temperature-dependent resistance for samples S_H_ and S_L_ with and without light. For the sample S_H_, the resistance keeps increasing with decreasing temperature in the dark, significantly below 100 K, where the resistance increases rapidly with temperature, showing semiconducting behavior. When exposed to light, the sample S_H_ exhibits a different resistance behavior, metallic above ~55 K and semiconducting below ~55 K. To rule out the thermal effects from irradiation laser at low temperatures, another sample S_L_, which shows metallic behavior from 300 K to 20 K in the dark, was also investigated. Under the same illumination conditions, sample S_L_ shows a slight reduction in resistance in the entire temperature range, implying that the apparent decrease in resistance after illumination of sample S_H_ is not caused by thermal effects. These results confirm that visible light can induce a metallic state in the semiconducting state of KTO.

Temperature-dependent photoresponse (*TPR*) is defined as [43]:

*TPR* = (*R_d_* − *R_l_*)/*R_l_* × 100%, (1)

where *R_d_* and *R_l_* are the resistance of the sample in the absence and presence of light. As shown in Figure 2b, the *TPR* of sample S_L_ does not change significantly from room temperature to 20 K, remaining essentially near 100%. In contrast, sample S_H_ shows a giant increment, especially at low temperatures. At 20 K, the resistance of sample S_H_ drops from 10^14^ Ω in the dark to around 10^4^ Ω under light, resulting in a huge *TPR* value of 10^12^%, suggesting that visible light has a more noticeable effect on the 2DEG at the KTO surface with higher resistance values. The significant difference between the two samples may be related to their different carrier densities. In general, the photogenerated carriers in 2DEG mainly come from the in-gap state-related transition in KTO. Considering that the photon energy in the current experiment (∼3.1 eV) is smaller than the band gap of KTO crystal (∼3.6 eV) [46], light illumination can only excite the in-gap states in KTO. When irradiated with the same laser power, the amount of photogenerated carriers should be the same for the two samples, supposing the recombination rates of carriers in the two samples are the same. Accordingly, the contribution of photocarriers to the total conductivity increases significantly in samples with lower carrier concentrations. Thus, the following experiments concentrate on the high-resistance sample S_H_ to better investigate the photoelectric response in the electron gas.

### 3.3. Temperature-Dependent Optoelectronic Properties with Different Laser Powers

**Figure 3:**
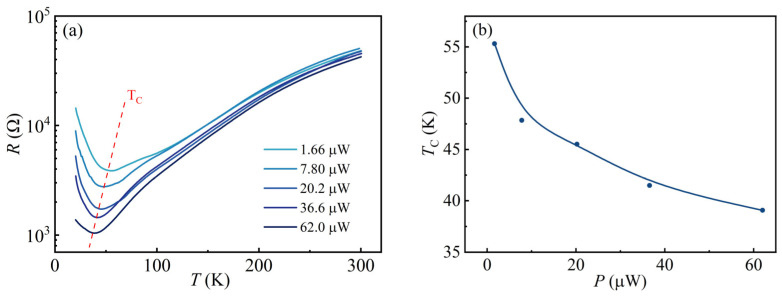
(**a**) Resistance as a function of temperature for 2DEG at the KTO surface, measured when the sample is exposed to light with different powers but a constant wavelength of 405 nm. Labels in the figure mark the power of the light. The red dashed line is *T_C_*; (**b**) *T_C_* as a function of laser power. Solid lines are guides for the eye.

Considering the obvious suppression of the semiconducting state of the electron gas at the KTO surface by visible light, it is necessary to investigate this phenomenon in more detail. It is supposed that different laser powers might have different effects on the transition temperature of 2DEG at the KTO surface. Figure 3a shows the resistance of 2DEG at the KTO surface as a function of temperature (20–300 K), measured using different power levels but with a constant wavelength of 405 nm to irradiate the S_H_ surface. It can be seen that the sample shows an apparent photo-conductivity behavior at all power levels. First, the resistance of the electron gas at the KTO surface at different laser powers decreases rapidly. Then, it increases slowly with increasing temperature, a metal-to-semiconductor transition occurs, and the transition temperature *T_C_* gradually moves to lower temperatures with increasing laser power. Figure 3b shows the variation in the transition temperature versus laser power. Obviously, the laser power can affect the transition temperature; as the laser power increases from 1.66 μW to 62.0 μW, the *T_C_* decreases from a starting value of 55 K to approximately 40 K. The laser-power-dependent transition temperatures extracted from Figure 3a are shown in Figure 3b. With the increase in light power, the sample resistance and *T_C_* monotonically decrease over the measurement temperature range, which may be attributed to the fact that the photogenerated carrier production rate is higher than the recombination rate under increasing laser power conditions, resulting in a gradual increase in the number of photogenerated carriers. It can be expected that if the sample surface is irradiated at a sufficiently high laser power, the semiconducting state 2DEG at the KTO surface in the low-temperature region will completely change to the metallic state.

### 3.4. Laser Power-Dependent Photoresponse at Low Temperatures

**Figure 4:**
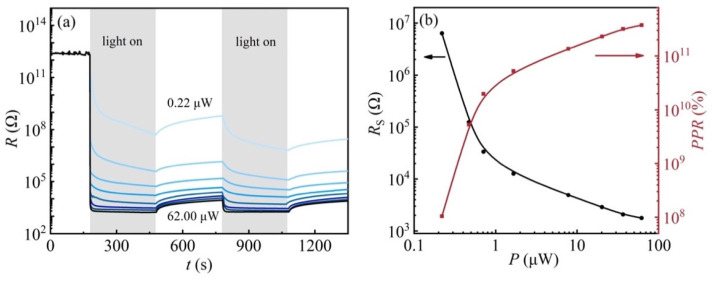
(**a**) Resistance response to the light illumination (λ = 405 nm), measured at a fixed temperature of 20 K, the blue lines from top to bottom denote the laser powers of 0.22 μW, 0.47 μW, 0.71 μW, 1.66 μW, 7.80 μW, 20.2 μW, 36.6 μW, and 62.0 μW, respectively; (**b**) The solid black line shows the saturation resistance as a function of laser power; the solid red line shows the optical response as a function of laser power.

From Figure 3a, a significant difference in the resistance of the sample at low temperatures was found when irradiated by different laser powers. To further investigate the effects of laser power on the resistance of 2DEG at the KTO surface at low temperatures, photoresponse experiments with different laser powers were carried out on sample S_H_ at 20 K. Figure 4a shows the sample resistance as a function of time during 405 nm light illumination in the power range between 0.22 and 62.0 μW. The sample was first illuminated for 300 s (light “ON”). Afterward, the light was turned off for 100 s (light “OFF”) to allow the resistance to reach a reasonably stable value, and then the “ON” and “OFF” processes were repeated once. The laser power has a great influence on the resistance of the 2DEG at the KTO surface. The change in the resistance of the 2DEG at the KTO surface becomes larger with increasing light intensity. The resistance of the sample is above 10^12^ Ω in an insulating state at 20 K in the dark and decreases down to 10^3^ Ω rapidly when irradiated with 62 μW light. It recovers more slowly after light cessation, showing an apparent persistent photoconductive behavior. In addition, the resistance shows a slow relaxation behavior under illumination with smaller laser power (0.22~7.80 μW), which can be attributed to its lower photo-generated carrier production rate, resulting in a gradual decrease in the resistance when the laser is turned on. Under the maximum laser power of 62.0 μW irradiation, the sample resistance decreases by nine orders of magnitude and can reach the saturation value instantaneously, indicating its potential application in photoelectric switching devices. 

To reveal the influences of laser power on the sample resistance, saturation resistance *R_s_* and the optical responsivity rate as a function of laser power are shown in Figure 4b, respectively. Here, the photoresponse rate (*PPR*) is defined as:

*PPR* = (*R_d_* − *R_s_*)/*R_s_* × 100%, (2)

where *R_s_* is the saturation resistance (minimum resistance under illumination). As shown in Figure 4b, applying a higher laser power results in a lower saturation resistance, and at laser powers of 1 μW or more, the effect of laser power on the saturation resistance becomes less critical, which is almost the same under illumination with different laser powers. The change trend of the *PPR* is the opposite of the *R_s_*. In addition, the increase in *PPR* with laser power is more significant when it is below 1 μW, which can be attributed to its lower recombination rate of photogenerated carriers.

### 3.5. Wavelength-Dependent Photoresponse at Different Temperatures

**Figure 5:**
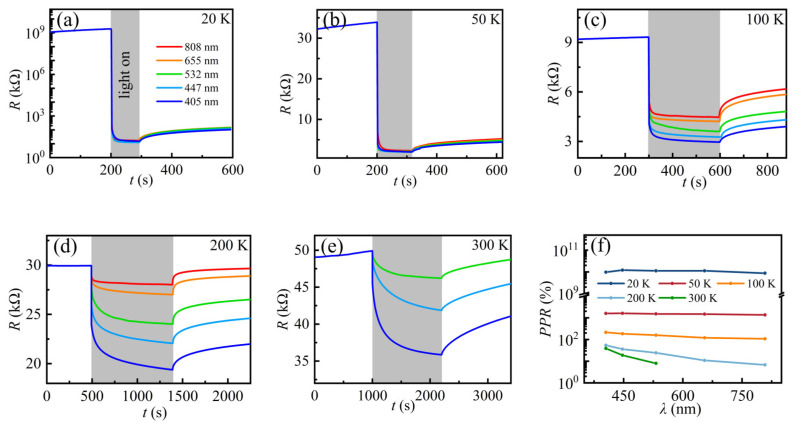
(**a**–**e**) Resistance response to light illumination, measured at different temperatures. The laser wavelengths of 405 nm, 447 nm, 532 nm, 655 nm, and 808 nm correspond to powers of 1.66 μW, 18.4 μW, 34.9 μW, 46.9 μW, and 49.9 μW, respectively. (**f**) *PPR* as a function of wavelength at different temperatures. The solid lines are guides for the eye.

The wavelength-dependent photoelectrical properties are investigated to further explore the photoresponse in electron gas at the KTO surface. Figure 5a–e show the photoresponse of 2DEG at the KTO surface under light irradiation with different wavelengths of the sample S_H_ at 20 K, 50 K, 100 K, 200 K, and 300 K, respectively. Upon decreasing the temperature, the optical response of the sample becomes larger, and the response time becomes shorter. At 300 K, when the laser wavelength is not more than 532 nm, the sample resistance decreased slowly when the light is turned on and cannot reach saturation even after 1200 s illumination, while the resistance hardly changed under 808 nm and 655 nm, consistent with the previous results in Ar^+^-bombarded KTO [41], which can be ascribed to their lower photon energy. When the sample is irradiated with an 808 nm and 655 nm laser, the energy obtained by the photogenerated carriers is not sufficient for them to reach the VBM, considering the enhanced electron scattering at elevated temperatures. In addition, the photoinduced slower relaxation process at 300 K provides another proof of the stronger electron scattering at high temperatures. Therefore, the sample shows no response to lasers at the two wavelengths, while the photons of other wavelengths are more energetic and can excite electrons from the in-gap band to the CBM. Normally, in the case of irradiation with the same photons, the shorter the wavelength of the laser, the higher the energy of the photon. Consequently,, more electrons can arrive at the CBM, showing a stronger photoresponse. When the temperature is down to 20 K, the sample resistance drops instantaneously from 10^12^ Ω to 10^4^ Ω, achieving a change of eight orders of magnitude. In addition, the effects of laser wavelength on the optical response of the sample S_H_ becomes weaker with decreasing temperature. At temperatures above 50 K, the saturation resistance *R_S_* decreases with decreasing laser wavelength. However, the effects of light wavelengths on the resistance change are negligible, and the saturation resistance *R_S_* was almost the same below 50 K.

A further interesting observation is that the sample shows a slow photoresponse at temperatures above 200 K. When illuminated light for 20 min, the resistance at 300 K is far from *R_m_*. Here, *R_m_* means the minimum resistance when exposed to a fixed laser power with enough time. This is very similar to our previous results of 2DEG at the STO surface [44]. By comparing the photoresponse of different temperatures, it can be concluded that temperature plays a key role in the photoinduced relaxation process, which becomes faster with decreasing temperature. At temperatures below 50 K, the photoresponse is very fast, similar to a band–band excitation. Considering that both the intrinsic band structure and the spatial distribution of the in-gap states cannot change with temperature, temperature-related electron scattering is responsible for the observed slow relaxation process at higher temperatures (above 100 K). 

Based on the data in Figure 5a–e, Figure 5f shows the wavelength-dependent photoresponse *PPR* of the samples at different temperatures. It can be observed clearly that the light wavelength has a significant effect on the photoresponse when the temperature is above 100 K. In comparison, at temperatures 20–50 K, the photoresponse is almost the same even when the sample is irradiated under different wavelengths. Therefore, compared to the wavelength, the photoresponse of the 2DEG is more sensitive to the laser power at low temperatures, especially at 20 K.

It is well known that oxygen vacancies play a vital role in the conductivity of oxide 2DEG. For the 2DEG at the KTO surface obtained by Ar ion bombardment, the bombardment time determines the amount of oxygen vacancies and hence the electron doping level [47]. It has been well recognized that the introduction of oxygen vacancies in the KTO surface results in a broad in-gap state in KTO located at 1.63 and 0.87 eV below the CBM [46]. In the study, the energy of all photons (1.0~3.06 eV) is smaller than the band gap of KTO, suggesting that the in-gap band associated with oxygen vacancies plays a key role in the photoresponse of the 2DEG at the KTO surface. After photon absorption, the electrons within the in-gap band are promoted to the conduction band of KTO close to the surface. Hence, more electrons participate in the conductivity. As a result, the resistance decrease caused by light illumination is mainly from the increased carrier amounts in the conduction band. At temperatures below 50 K, when the effects of thermal disturbance (TD) can be ignored, an almost direct promotion from the in-gap state to the CBM occurs since the photon energy is higher than the in-gap state (0.87 eV below the CBM), resulting in a fast and wavelength-independent transition process. With increasing temperature, the TD effect becomes stronger gradually and the photocarriers lose more and more energy during the transition. As a result, the time it takes for the photocarriers to reach the CBM becomes longer, so an increasingly pronounced relaxation process is observed when the temperature is above 100 K. When it rises to 300 K, the energy for the photocarriers from 808 nm and 655 nm light absorption is not enough to reach the CBM; thus, no photoconductivity response is observed for the two wavelengths. 

**Figure 6:**
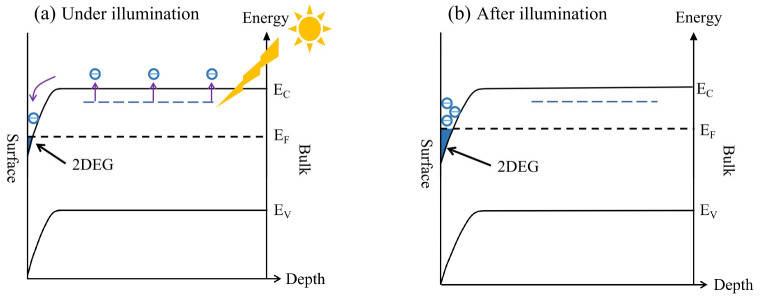
Charge transfer mechanism of the 2DEG at the KTO surface under (**a**) and after (**b**) visible light illumination. The purple arrows denote the direction of motion of the electrons, and blue regions denote the electron accumulation layer, namely the 2DEG layer.

The onset of metallic conductivity in the 2DEG at the KTO surface can be attributed to the increased carriers after light illumination. Figure 6a,b show the schematic diagrams of the energy-band structures under and after light illumination. As shown in Figure 6, there are partially occupied defect states related to oxygen vacancies in the band gap region of KTO. Surface barriers are also formed in the process of surface 2DEG formation [48]. When exposed to light with photon energy above 0.87 eV, the in-gap states will be excited, and the photogenerated electron/hole pairs in the 2DEG are spatially separated from the parent donor defects and brought into the surface potential well, where the accumulation of electrons occurs on the surface of the KTO, resulting in the semiconductor-to-metal transition at temperatures above 50 K. At temperatures below 50 K when the original electron concentration is too low, the 2DEG still maintains its original semiconducting state, even though a lot of photogenerated carriers participate in the conductivity. Indeed, as observed by Zeeshan et al. [43], the temperature-dependent transition is a result of visible-light-induced in-gap band promotion. After the removal of the light, most of the photoexcited electrons cannot easily return to their initial positions due to the resistance exerted by the macroscopic potential barriers, showing an obvious persistent photoconductivity effect. Consistent with the results observed by Goyal et al. in which a defect-related photoresponse is persistent and shows slow relaxation behavior [42], the slower relaxation process observed at higher temperature can be ascribed to oxygen vacancy-related photoexcitation.

## 4. Conclusions

In summary, we have demonstrated that the semiconducting state in the 2DEG at the KTO surface can be suppressed by visible light illumination. With 405 nm light irradiation, semiconducting 2DEG can be transited into the metallic state in the temperature range of 300–55 K. By increasing the laser power, the transition temperature can be lower. Similar to previous reports, the original state cannot recover immediately after the light has been switched off for a long time, showing persistent photoconductivity. All the photon energy is smaller than the energy gap of KTO, indicating the dominant role of the in-gap states in the photoexcitation process. Our results provide a deeper understanding of the photoinhibition of the semiconducting state of 2DEG at the KTO surface and help to explore the photoinduced modulation effect of 2DEG at the KTO surface. 

## Data Availability

The data presented in this study are available from the corresponding author upon reasonable request.
